# Life expectancies and incidence rates of patients under prolonged mechanical ventilation: a population-based study during 1998 to 2007 in Taiwan

**DOI:** 10.1186/cc10128

**Published:** 2011-04-01

**Authors:** Mei-Chuan Hung, Hsin-Ming Lu, Likwang Chen, Fu-Chang Hu, Soa-Yu Chan, Yuan-Horng Yan, Po-Sheng Fan, Ming-Shian Lin, Cheng-Ren Chen, Lu-Cheng Kuo, Chong-Jen Yu, Jung-Der Wang

**Affiliations:** 1 Institute of Occupational Medicine and Industrial Hygiene, College of Public Health, National Taiwan University, No 17, Xuzhou Road, Taipei 100, Taiwan; 2 Institute of Population Health Sciences, National Health Research Institutes, 35 Keyan Road, Zhunan 350, Taiwan; 3 Institute of Public Health, School of Medicine, National Yang-Ming University, No. 155, Section 2, Linong Street, Taipei 112, Taiwan; 4 Institutes of Clinical Medicine, College of Medicine National Taiwan University, No. 1, Jen Ai Road, Taipei 100, Taiwan; 5 International Harvard Statistical Consulting Company, 57 Chongqing South Road, Section 1, 7F-11, Taipei 100, Taiwan; 6 Department of Medical Research, National Taiwan University Hospital, No. 7, Chung Shan S. Road, Taipei 100, Taiwan; 7 Department of Internal Medicine, Chia-Yi Christian Hospital, 539 Jhongsiao Road, Chiayi 600, Taiwan; 8 Department of Medical Research, Chia-Yi Christian Hospital, 539 Jhongsiao Road, Chiayi 600, Taiwan; 9 Department of Internal Medicine, National Taiwan University Hospital, No. 7, Chung Shan S. Road, Taipei 100, Taiwan; 10 Department of Public Health, National Cheng Kung University College of Medicine, No. 1, University Road, Tainan 701, Taiwan; 11 Departments of Internal Medicine and Environmental and Occupational Medicine, National Cheng Kung University Hospital, No. 138, Sheng Li Road, Tainan 704, Taiwan

## Abstract

**Introduction:**

The present study examined the median survival, life expectancies, and cumulative incidence rate (CIR) of patients undergoing prolonged mechanical ventilation (PMV) stratified by different underlying diseases.

**Methods:**

According to the National Health Insurance Research Database of Taiwan, there were 8,906,406 individuals who obtained respiratory care during the period from 1997 to 2007. A random sample of this population was performed, and subjects who had continuously undergone mechanical ventilation for longer than 21 days were enrolled in the current study. Annual incidence rates and the CIR were calculated. After stratifying the patients according to their specific diagnoses, latent class analysis was performed to categorise PMV patients with multiple co-morbidities into several groups. The life expectancies of different groups were estimated using a semiparametric method with a hazard function based on the vital statistics of Taiwan.

**Results:**

The analysis of 50,481 PMV patients revealed that incidence rates increased as patients grew older and that the CIR (17 to 85 years old) increased from 0.103 in 1998 to 0.183 in 2004 before stabilising thereafter. The life expectancies of PMV patients suffering from degenerative neurological diseases, stroke, or injuries tended to be longer than those with chronic renal failure or cancer. Patients with chronic obstructive pulmonary disease survived longer than did those co-morbid with other underlying diseases, especially septicaemia/shock.

**Conclusions:**

PMV provides a direct means to treat respiratory tract diseases and to sustain respiration in individuals suffering from degenerative neurological diseases, and individuals with either of these types of conditions respond better to PMV than do those with other co-morbidities. Future research is required to determine the cost-effectiveness of this treatment paradigm.

## Introduction

The number of patients who require prolonged mechanical ventilation (PMV) is rapidly increasing worldwide, apparently due to aging, a greater number of co-morbidities, and the increasing availability and effectiveness of this new technology [1-3]. The fact that many patients require continued respiratory care after being transferred into a rehabilitation facility creates a tremendous financial burden [3,4]. Furthermore, there is often a gap between families' unreasonably optimistic expectations and clinicians' professional judgement. This gap frequently results in difficulties arriving at consensus clinical decision-making [5]. In many cases, these challenges are not easily resolved. These issues are exacerbated by the lack of evidence regarding expected survival times for different subgroups of patients, especially for those suffering from multiple co-morbidities.

The National Health Insurance (NHI) of Taiwan has implemented a system of comprehensive coverage for various healthcare services, including maintenance haemodialysis and chronic respiratory care. The NHI was first established in 1995 and has been extended to cover over 99% of the citizens of Taiwan [6,7]. In 1998, the Bureau of the NHI drafted a prospective payment programme to encourage integrated care for mechanically ventilated patients, which was implemented in July 2000 [8]. After several revisions, this programme ultimately covered mechanical ventilator care in the following settings: ICUs (acute stage, <21 days), respiratory care centres (a subacute stage for weaning training, up to 42 days), respiratory care wards (a chronic stage or long-term care), and homecare services (a stable stage during which the patient is cared for directly by family caregivers). The rising number of patient-days for mechanical ventilation usage during 1997 to 2004 increased the financial burden of the NHI [9].

Similar to the case in western countries [5], discrepancies frequently exist in Taiwan between a family's initial expectations and their physician's professional judgement. These discrepancies impair communication among patients, their families, and healthcare workers for clinical decision-making before and throughout the course of installing mechanical ventilation. There is thus a need to estimate the incidence rates and life expectancies for PMV patients with various diagnoses. Accurate prognoses are essential to propose and establish a sustainable national policy and to facilitate communication among different stakeholders. To examine the above issues, we collected a random sample from the national database and compared age-specific incidence rates, cumulative incidence rates (CIRs), median survival, and life expectancies of PMV patients stratified according to their underlying diseases.

## Materials and methods

### Study population, datasets, and calculation of age-specific and cumulative incidence rates

The present study was approved by the Institutional Review Board of the National Taiwan University Hospital, which also waived the requirement for obtaining informed consent because the study was conducted on a secondary database with encrypted identification numbers. The reimbursement data file obtained from the NHI of Taiwan was transformed into a research database by the National Health Research Institutes (in Chunan, Taiwan) [10]. The identification numbers of all individuals in the reimbursement data file were encrypted to protect their privacy. These files contained detailed demographic data (including birth date and sex) and information regarding the healthcare services provided for each patient, including all payments for outpatient visits, hospitalisations, prescriptions, diagnoses, and intervention procedures. The data for each inpatient hospitalisation included up to five diagnoses, which were coded according to the International Classification of Diseases (Ninth Revision) and the date of each prescription or procedure. In total, 8,906,406 individuals had undergone invasive or non-invasive respiratory care at least once during the period from 1997 to 2007. This number corresponds to approximately 29.4% of the entire insured population. Because the government has established guidelines stating that no more than 10% of all data can be drawn for research, we applied for a random sample of these patients with a 3.4:1 ratio and enrolled subjects who had undergone mechanical ventilation for longer than 21 days.

**Figure 1:**
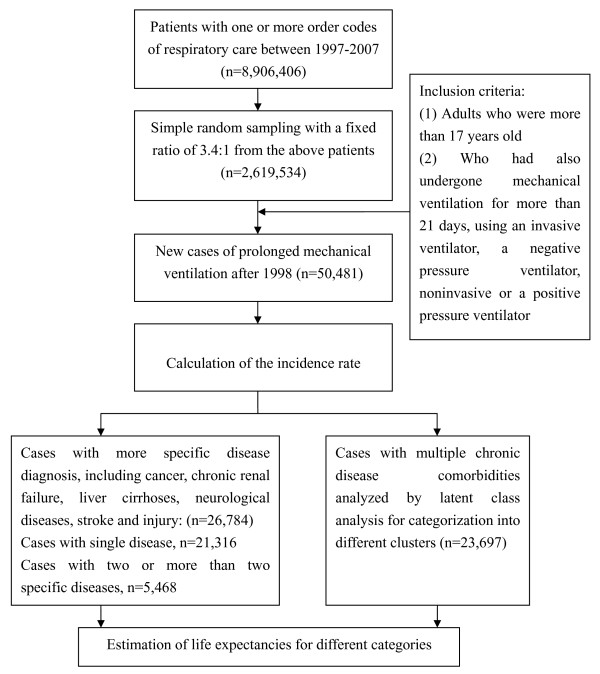
**Flow chart of the selection process used for the study cohort**.

According to the definition of PMV in Taiwan [8], we included patients over the age of 17 who had undergone either invasive or non-invasive mechanical ventilation, with negative or positive pressure ventilators for at least 21 consecutive days in the ICU or the respiratory care centre. To ensure that all of the patients were incident cases, we excluded all prevalent cases found in 1997 and began the collection in 1998, as illustrated in Figure 1. The calendar year-specific and age-specific incidence rates were determined by taking the number of new cases of PMV patients in that stratum, multiplying by the sampling factor of 3.4, and then dividing the resulting value by the number of individuals within the specific stratum obtained from the census of the Ministry of the Interior in Taiwan [11]. The CIR (cumulative incidence rate) formula was calculated as follows [12]:

where IR*_i _*represents the age-specific incidence rate and Δ*t_i _*indicates the range of each age stratum. We calculated the CIR_17 to 85_, which estimates the likelihood that an average person in Taiwan would require PMV assuming that he or she lives to the age of 85.

### Categorisation of patients for estimation of life expectancies

All recruited subjects were followed until the end of 2007 to determine whether they were alive, deceased, or censored. Because patients who had undergone PMV usually suffered from a combination of multiple co-morbidities and five major diagnoses can be retrieved for each PMV patient prior to and closest to the first day, we developed a strategy to identify different homogeneous groups to make more accurate estimates of life expectancy.

First, we excluded specific categories with extremely low frequencies, including HIV infection (*n *= 27) and complications during pregnancy/childbirth/perinatal period (*n *= 240). Second, people with major diagnoses that may cause premature mortality were stratified and analysed separately, including cancer, end-stage renal disease, liver cirrhosis, multiple sclerosis or degenerative neurological diseases, Parkinson's disease, and injury or poisoning, as each group shares major common characteristics that predict mortality. Third, because the vast majority of patients suffered from a combination of multiple chronic diseases that may cause premature mortality, such as diabetes mellitus, coronary and/or heart failure, hypertension, respiratory system and/or urinary tract infections, acute renal failure, septicaemia with and without shock, and so forth, we conducted latent class analysis (LCA) for these cases to categorise them into clusters or specific homogeneous groups for estimations of life expectancies. During this process, we grouped several closely related diagnoses together and converted their original International Classification of Diseases (Ninth Revision) codes into Clinical Classifications Software codes [13] so that sufficient numbers could be obtained for survival analysis. Following the above direction, the International Classification of Diseases (Ninth Revision) codes for septicaemia included 0031, 0202, 0223, 0362, all subcategories of 038, and 7907 (bacteraemia); those for shock included all subcategories under code 785.

### Statistical analysis

Binary and categorical variables were summarised using frequency counts and percentages. Continuous variables that were distributed normally are presented as means.

### Latent class analysis

To determine the underlying causes that were more likely to lead to PMV, we applied LCA to group separate co-morbidity diagnoses into no more than 10 clusters of in-patients who had undergone PMV. Because pneumonia and respiratory failure are the most common reasons for mechanical ventilation, these conditions were not included in this model. The analysis resulted in an LCA model consisting of 32 broad diagnosis categories, which included chronic diseases that had been previously classified into 260 categories by Clinical Classifications Software.

LCA assumes that responses are conditionally independent within classes after accounting for class membership [14]. In other words, LCA allows for the grouping of the PMV patients into several relatively homogeneous clusters of diagnosis patterns. In constructing the model, each cluster or class was named after the major disease (that is, with the highest prevalence or likelihood) present within each age strata.

Akaike Information Criteria were used to assess the goodness of fit of the model [15]. Lower Akaike Information Criteria statistics were considered to indicate a better statistical fit of the model to the data. If any single category exhibited a prevalence approaching 100% for a given condition across different age groups, then we assumed that these conditions could be reclassified into groups with specific diseases, and life expectancy estimations were conducted separately. Throughout this process, we found that only stroke could be further separated from the groups of multiple co-morbidities, and thus the life expectancy estimation for stroke patients was performed independently. SAS statistical software (version 9.1; SAS Institute, Cary, NC, USA) and R statistical software (version 2.10.1; R Foundation for Statistical Computing, Vienna, Austria) were used for the data analyses.

### Estimation of life expectancy

Each new patient who fulfilled the definition of PMV was followed beginning on the first day of PMV treatment and continuing until he/she was deceased or censored on 31 December 2007. The median survival, or the time at which only one-half of the patients within a given category were still alive, was estimated by the Kaplan-Meier method. In general, most patients did not survive longer than 1 to 3 years, although some patients did exhibit a longer survival time. All patients survived the initial 21 days of treatment by mechanical ventilation, and the survival times reported here exclusively refer to survival duration thereafter. The lifetime survival of PMV patients (up to 300 months when excluding those older than 85 years) was thus obtained using a linear extrapolation of a logit-transformed curve of the survival ratio between the PMV and an age-matched and gender-matched reference population generated by the Monte Carlo method from the life table of the general population of Taiwan. The detailed method and mathematical proof assuming a constant excess hazard have been described in our previous reports [16-20]. To facilitate the computation we used ISQoL, a software program that was built based on the *R *statistical package for lifetime expectancy estimation and 300-month extrapolation (excluding those older than 85 years) and can be downloaded for free [21].

### Validation of the extrapolation method for survival functions

Empirical PMV data from the National Health Research Institutes provided us with an opportunity to validate the actual performance of our semiparametric method of estimation. We therefore selected subcohorts of patients beginning on the first day that they received PMV between 1998 and 2001. We assumed that these cohorts were only followed until the end of 2001 and then extrapolated these results to the end of 2007. We compared our predictions with the Kaplan-Meier estimates of the direct follow-ups from 1998 to 2007. Assuming that the Kaplan-Meier estimates are the gold standard, we calculated the relative biases for subcohorts stratified by different underlying diseases and co-morbidities [22]. The relative biases were computed to compare the differences in values between the Kaplan-Meier estimates and the Monte Carlo extrapolation method.

## Results

### Basic characteristics of the prolonged mechanical ventilation cohort

A total of 50,481 new patients with PMV were included during the study period (40% female, mean age 72 ± 14.5 years, median survival 0.37 years, and overall life expectancy 2.68 years). If we counted only the primary diagnosis (out of a maximum of five diagnoses) for each patient, the top five primary diagnoses were acute respiratory failure (15%), pneumonia (12%), intracerebral haemorrhage (5%), septicaemia (3%), and chronic airways obstruction (2%). The tracheotomy rate was 60.1%, which reflects the ethnic Chinese cultural tradition that typically avoids additional traumatic wounds if a patient is expected to pass away soon.

### Trends of age-specific incidence rates and cumulative incidence rates over time

After the NHI began to reimburse long-term usage of mechanical ventilation to relieve the congested intensive care ward in 1998, the incidence rate started to rise and showed an increased trend with older age (Table 1). In the groups aged 65 to 74 years, 75 to 84 years, and older than 85 years, increased incidence rates of 76%, 88%, and 119%, respectively, were observed from 1998 to 2004, followed by a slight drop after 2005. The CIR (17 to 85 years) increased from 0.103 in 1998 to 0.183 in 2004 and then decreased to 0.145 in 2007.

**Table 1:** Age-specific incidence rates (per 100,000 person-years), and CIR of patients under prolonged mechanical ventilation

Age group (years)	1998	1999	2000	2001	2002	2003	2004	2005	2006	2007
Number of new cases	9,296	12,651	12,913	15,660	17,731	19,737	21,818	21,692	20,414	19,723
17 to 34	5.1	6.1	5.5	5.8	6.1	8.4	9.9	9.2	9.4	9.9
35 to 44	10.1	10.8	11.8	14.5	14.4	15.4	19.2	17.4	19.8	18.1
45 to 54	21.8	33.3	29.3	32.4	37.2	42.0	40.5	43.5	39.5	39.7
55 to 64	78.2	101.1	92.2	102.5	111.6	118.9	129.6	119.8	101.7	93.0
65 to 74	224.0	296.5	284.5	329.2	361.9	379.8	393.9	369.2	331.8	306.4
75 to 84	622.0	817.2	814.6	967.2	1,045.5	1,072.3	1,166.4	1,036.0	1,004.6	909.2
≥85	1,182.0	1,536.0	1,702.6	2,064.1	2,253.0	2,563.9	2,584.0	2,554.0	2,161.0	2,046.0
CIR	0.103	0.133	0.132	0.153	0.165	0.173	0.183	0.170	0.159	0.145

CIR, cumulative incidence rate (aged 17 to 85 years).

### Life expectancies of prolonged mechanical ventilation patients with specific underlying diseases

The median survival and life expectancies of PMV patients with different diseases are summarised in Table 2. Although median survival for most categories was <1 year, many patients showed life expectancies longer than 2 to 3 years, indicating that some patients survived relatively long periods of time. The median survival and life expectancies of PMV patients with degenerative neurological disease, stroke, or injuries were generally longer than those with chronic renal failure or cancer. When a patient contracted both cancer and chronic renal failure, the median survival durations and life expectancies were the shortest. Patients with stroke were initially included in the LCA because of the presence of multiple co-morbidities, but a distinctive category of 100% prevalence of stroke consistently appeared across different age strata. We therefore separated this group and estimated the associated life expectancies for different age strata, as summarised in Table 2.

**Table 2:** Demographic characteristics and survival of patients undergoing prolonged mechanical ventilation stratified by different underlying diseases

	Number of cases	Mean age (SD)	Female (%)	Median survival (years)	Life expectancy (SE) (years)
**Cases with single specific disease**	21,316	69 (15)	38	0.35	3.40 (0.09)
Cancer	5,367	70 (14)	33	0.17	1.51 (0.13)
Chronic renal failure	2,032	73 (12)	51	0.78	1.36 (0.16)
Liver cirrhosis	1,478	65 (17)	35	0.19	3.59 (0.33)
Multiple sclerosis or degenerative nervous system conditions	378	65 (17)	39	0.89	4.05 (0.64)
Parkinson's disease	341	79 (7)	36	0.85	2.06 (0.30)
Stroke	6,765	70 (13)	42	0.72	3.38 (0.15)
Aged <64 years	1,955	53 (9)	35	1.65	5.21 (0.39)
Aged 65 to 74 years	1,818	70 (3)	43	0.77	2.98 (0.17)
Aged 75 to 84 years	2,176	79 (3)	44	0.56	2.09 (0.13)
Aged >85 years	816	88 (3)	54	0.39	1.68 (0.13)
Intracranial and/or spinal cord injury or poisoning	4,955	65 (19)	34	1.06	6.27 (0.24)
Aged <64 years	1,949	45 (14)	26	6.20	10.20 (0.49)
Aged 65 to 74 years	1,116	70 (3)	39	0.82	3.77 (0.22)
Aged 75 to 84 years	13,66	79 (3)	35	0.47	2.67 (0.19)
Aged >85 years	524	88 (3)	48	0.33	1.82 (0.13)
**Cases with more than two specific diseases**	4,772	68 (15)	39	0.32	2.96 (0.13)
Cancer and chronic renal failure	165	71 (11)	44	0.14	1.21 (0.45)
Cancer and others	1,609	70 (14)	35	0.19	1.88 (0.22)
Chronic renal failure and others	743	70 (13)	50	0.21	1.71 (0.28)

SD, standard deviation; SE, standard error of the mean.

### Life expectancies of age-specific clusters in prolonged mechanical ventilation patients with multiple co-morbidities

Among the 23,697 PMV patients with multiple co-morbidities, the latent class model usually yielded three or four clusters, including heart diseases, septicaemia/shock, chronic obstructive pulmonary diseases, and/or others (for example, urinary tract infections), as summarised in Table 3. Diabetes mellitus seemed to be the most frequent co-morbid disease among all clusters because the prevalence rates were all above 14.5%. The life expectancy and median survival of PMV patients with chronic obstructive pulmonary disease (COPD) were generally longer than those of other clustered groups, especially those with septicaemia/shock. This trend continued until the age of 85, after which PMV patients with different underlying co-morbidities seem to show similar outcomes.

**Table 3:** Clusters of different co-morbidities categorised by latent class analysis in patients with prolonged mechanical ventilation

	Age <64 years (*n *= 3,520)				Age 65 to 74 years (*n *= 5,397)			Age 75 to 84 years (*n *= 9,747)				Age >85 years (*n *= 5,033)		
				
	Class 1	Class 2	Class 3	Class 4	Class 1	Class 2	Class 3	Class 1	Class 2	Class 3	Class 4	Class 1	Class 2	Class 3
	
	Heart disease	SP/shock	UTI/SP	COPD/other	Heart disease	SP/shock	COPD/other	Heart disease	SP/shock	COPD/other	Respiratory disease	Heart disease	SP/shock	COPD/other
Number of cases	616	919	197	1,788	1,074	1,824	2,499	1,404	2,856	4,142	1,345	870	1,359	2,804
**Prevalence of co-morbidity (%)**														
Septicaemia	11.3	62.9	72.2	6.7	10.6	66.1	5.5	11.8	72.8	11.0	7.2	10.2	79.1	11.7
Diabetes mellitus	35.5	26.8	26.1	19.7	39.7	26.1	26.5	27.1	20.5	22.6	17.2	18.6	14.5	14.6
Hypertension	17.9	4.9	9.8	8.3	20.8	6.8	13.9	16.1	4.8	17.3	12.5	15.4	3.3	15.2
AMI/coronary atherosclerosis	39.9	3.9	2.3	2.4	43.9	4.6	5.2	42.8	5.5	6.6	4.0	42.8	5.6	4.0
COPD	6.5	3.2	0.5	17.9	11.0	11.0	33.9	18.2	12.4	39.6	29.6	24.9	12.7	33.0
Other respiratory disease	21.8	23.7	13.8	30.1	22.3	18.1	26.7	20.9	17.2	0	100	24.6	19.4	25.9
Acute renal failure	12.2	20.6	9.5	4.8	12.7	16.4	4.5	12.1	16.7	4.2	5.3	8.7	15.2	5.6
UTI	5.3	0	100	14.8	5.7	25.1	21.1	11.7	28.0	24.3	21.1	16.1	30.6	29.0
Shock	11.2	39.6	33.1	2.7	10.3	39.1	2.7	8.9	38.5	4.9	3.7	7.3	41.6	5.0
Heart failure	42.2	3.2	2.6	2.4	37.0	5.8	4.8	50.1	7.2	7.7	7.4	50.5	8.3	7.7
Median survival (years)	0.80	0.34	0.88	1.64	0.39	0.23	0.55	0.29	0.21	0.95	0.38	0.32	0.20	0.35
Life expectancy (years) (SE)	5.09 (0.60)	4.51 (0.49)	4.82 (1.49)	5.25 (0.37)	2.55 (0.21)	2.14 (0.13)	2.56 (0.13)	1.86 (0.23)	1.66 (0.10)	2.12 (0.07)	2.18 (0.17)	1.48 (0.12)	1.12 (0.07)	1.52 (0.05)

AMI, acute myocardial infarction; COPD, chronic obstructive pulmonary disease; SE, standard error of the mean; SP, septicaemia; UTI, urinary tract infections.

### Validation results of the extrapolation method

The results obtained to validate our semiparametric method show that the relative biases were all below 20%. Among them, the relative biases of most PMV patients with a specific diagnosis ranged between 0.9 and 5.5%. Stroke patients were an exception and usually suffered from other co-morbidities. Patients with a combination of different diseases (or clusters) appeared less likely to fulfil the assumption of a constant excess hazard completely and resulted in greater relative biases, perhaps because they represent a relatively heterogeneous patient population. Nonetheless, the absolute differences between our estimates and those obtained using the Kaplan-Meier method were all below 0.25 life-years, except for the 65-year-old to 74-year-old multiple co-morbidity categories, which showed an absolute difference of 0.39 life-years.

## Discussion

To our knowledge, this is the first study to analyse a nationally representative PMV dataset to estimate the incidence rates, CIR, and life expectancies stratified by age and different clusters of diagnoses. Our findings showed that new cases of PMV increased significantly from 9,296 to 21,818 between 1998 and 2004. The age-specific incidence rates increased as people grew older, a result consistent with previous reports from scholars in the United States and Canada [1,2,4,23]. The highest age-specific incidence rate of PMV was observed in patients older than 85 years in Taiwan, however, and this rate was approximately four to five times higher than those reported in the United States [24]. We attempted to quantify the lifetime risk of PMV by calculating the CIR_17 to 85, _which increased from 0.103 to 0.145 between 1998 and 2007 (Table 1). This finding implies that an adult person in Taiwan who lives until the age of 85 has a 10 to 15% chance of requiring PMV. Given the resource-intensiveness of PMV, this issue requires special attention. When the Bureau of the NHI of Taiwan began to audit the quality of the integrated respiratory care system in 2003, including the rates of successful weaning, readmission, and nosocomial infection, the incidence of PMV appeared to stabilise and decreased slightly, as summarised in Table 1.

In the past, there has been a general lack of data regarding the life expectancies associated with different diagnoses for patients undergoing PMV. This has made it difficult for stakeholders to reach consensus clinical decisions regarding optimal treatment strategies. The issue becomes even more complicated when payment is provided via NHI or a third party. It is understandable that the patient and his/her family always expect successful weaning and good recovery, even after longer than 21 days of continuous mechanical ventilation or PMV. According to our previous study, however, most patients undergoing PMV survive only approximately 1.5 to 2 years, and approximately 62% of them suffer from cognitive impairments and poor quality of life. Accounting for these factors results in an overall quality-adjusted life expectancy of only 0.3 to 0.4 and 0.6 to 0.7 quality-adjusted life-years [25,26]. The present study therefore further provided crucial estimates of the median survival and life expectancies of patients undergoing PMV with different diagnoses or co-morbidities, as summarised in Tables 2 and 3. Table 2 shows that the life expectancies were shortest for PMV patients with chronic renal failure and cancer or any condition co-morbid with them, followed by Parkinson's disease and stroke. In contrast, the life expectancies for degenerative neurological diseases, liver cirrhosis, injuries, and poisonings were >3.6 years. When stratified by age categories, the median survival and life expectancies for PMV patients older than 85 years were <4.6 months and <21.8 months, respectively, which were also observed for all of the different types of co-morbidities (Tables 2 and 3). The above figures call into question the cost-effectiveness of current policies and should be considered by policy-makers and the public in discussions regarding the bioethics of PMV care, especially given the limited resources of the NHI in Taiwan. Although more and more countries have tried to implement the principle of universal coverage in their national health insurance plans [27], our results provide data highlighting the needed evidence for developing strategies of sustainable management.

Although previous studies have shown similar characteristics of multiple co-morbidities in PMV patients, these reports did not stratify patients into special clusters [1,9,24,28]. The LCA showed that the underlying co-morbidities associated with PMV could be largely classified into the major categories of heart diseases, septicaemia/shock, and COPD based on the high prevalence of each cluster. Overall, LCA indicates that the life expectancies generally decreased with older age. In particular, we found that approximately 50% of the PMV patients with a combination diagnosis of septicaemia and shock usually survived <4 months, and their life expectancies were usually shorter than those determined for the other clusters within the same age stratum. The generally longer survival time of PMV patients with COPD corroborated the hypothesis that the establishment of mechanical ventilation provides more direct access for clinicians to solve problems coming from the respiratory tract, while patients with other underlying diseases may not be improved significantly unless their underlying disorders were also resolved. This advantage disappeared in individuals over 85 years of age because a high proportion of these COPD patients also suffered from other major diseases, including urinary tract infection (29%) and other respiratory diseases (26%), as shown in Table 3.

Our study has several limitations. First, the database did not contain any information regarding the severity and/or actual clinical data of the PMV patients. We were therefore unable to further stratify these patients. Because they were all under PMV care for longer than 21 days, however, all of the patients were associated with extremely severe conditions, which resulted in a very short life expectancy and suggested that 10 years of follow-up time would be usually sufficient. Second, because the recorded diagnoses must fulfil all of the reimbursement regulations of the NHI, it is possible that some diagnoses are over-represented because they were more easily reimbursed. However, the NHI of Taiwan has offered a list of 30 major categories of catastrophic illnesses that are exempt from partial co-payments, and each has its specific diagnostic criteria to prevent any abuse [29]. For example, all types of malignant neoplasm do not require co-payments, and evidence of histopathology and/or cytology is generally required for diagnoses of cancer. A diagnosis of end-stage renal disease requires documentation of chronic kidney disease with an irreversible creatinine level >8 mg/dl, or creatinine level >6 mg/dl with diabetes mellitus as a co-morbid condition [30]. There are therefore strict criteria for almost all of the major diagnoses listed in Table 2. The potential selection bias for the common diseases listed in Table 3 is probably minimal because the 43 broad categories were collapsed from the 260 categories of Clinical Classifications Software codes [13], and LCA ensured that each category was as homogeneous as possible.

## Conclusions

The number of PMV patients in Taiwan has increased during the past decade. Patients with different underlying diseases showed different median survival and life expectancies. The establishment of mechanical ventilation directly targets problems of the respiratory tract and provides sustainable ventilation, which may improve the survival of patients with COPD or degenerative neurological diseases more than those with other underlying causes such as septicaemia/shock, heart failure, cancer, or end-stage renal disease. The advantages of PMV seem to decrease for the older patient, however, especially those aged over 85. The results also call for further evaluation of the cost-effectiveness and bioethics of such care in Taiwan, and highlight the need for early planning of resource allocation in any system of health insurance with universal coverage.

## Key messages

• The number of new patients undergoing treatment with PMV has increased rapidly during the past decade in Taiwan.

• The life expectancies of PMV patients with degenerative neurological diseases, stroke, or injuries/poisoning as their primary co-morbidity seemed to survive longer than those with chronic renal failure or cancer, or a co-morbidity with them.

• Among PMV patients with multiple co-morbidities, those with COPD as the major underlying co-morbidity seem to survive longer than patients with other co-morbidities, perhaps because this treatment specifically targets the respiratory tract, which is compromised in COPD. The benefits of PMV decrease for the older patient, especially those aged over 85.

## Abbreviations

CIR: cumulative incidence rate; COPD: chronic obstructive pulmonary disease; ICU: intensive care unit; LCA: latent class analysis; NHI: National Health Insurance; PMV: prolonged mechanical ventilation.

## Competing interests

The authors declare that they have no competing interests.

## Authors' contributions

M-CH was involved in the study design, data analysis, and manuscript preparation. H-ML was involved in sample storage and data analysis. LC was involved in the study design and manuscript revisions. F-CH was involved in the statistical analysis of the data and manuscript revisions. S-YC was involved in the statistical analysis of the data. Y-HY, P-SF, M-SL, L-CK and C-JY were involved in the study design and manuscript revisions. C-RC was involved in the overall study design and the preparation and revision of the manuscript. J-DW was involved in the overall study design and supervision, the data analysis, as well as the preparation and revision of the manuscript. All authors read and approved the final manuscript.
